# Interferon (IFN)-λ Takes the Helm: Immunomodulatory Roles of Type III IFNs

**DOI:** 10.3389/fimmu.2017.01661

**Published:** 2017-11-28

**Authors:** Ivan Zanoni, Francesca Granucci, Achille Broggi

**Affiliations:** ^1^Harvard Medical School, Division of Gastroenterology, Boston Children’s Hospital, Boston, MA, United States; ^2^Department of Biotechnology and Biosciences, University of Milano-Bicocca, Milan, Italy

**Keywords:** interferon lambda, dendritic cells, neutrophils, natural killer cells, type III interferon, viral infection, bacterial infections, fungal infection

## Abstract

Type III interferons (IFNs) (or IFN-λ) are the latest addition to the IFN family. Even though they share little protein homology with type I IFN, both exhibit remarkable functional similarities: each can be induced in response to viral infections, and both lead to Janus kinases (JAK) and signal transducer and activator of transcription (STAT) activation. The JAK/STAT pathway induces antiviral responses and IFN-stimulated gene transcription. However, despite the similarities in their effector functions with type I IFNs, IFN-λ also has a non-redundant role in protecting barrier organs: epithelial cells preferentially produce IFN-λ rather than type I IFNs; and interferon lambda receptor 1 (IFNLR1), the specific receptor for IFN-λ, is highly expressed on cells of epithelial lineage. Thus far, IFN-λ has been considered mainly as an epithelial cytokine, which restricts viral replication in epithelial cells and constitutes an added layer of protection at mucosal sites. However, it is now increasingly recognized that IFNLR1 is expressed broadly, and that immune cells such as neutrophils and dendritic cells also respond to IFN-λ. Moreover, in many *in vivo* models, IFN-λ modulates immune cell functions and thereby configures itself less as a cytokine that is only specific to the epithelium, and more as a cytokine that directly controls the inflammatory response at mucosal sites. Here, we critically review the recent literature on immune modulatory roles for IFN-λ, and distinguish between the direct and indirect effects of this IFN on immune cell functions in different inflammatory settings.

## Introduction

First described more than 60 years ago (1) interferons (IFNs) were the first family of cytokines to be discovered. Since then, IFNs have been extensively studied, and their presence is correlated with a number of immunological and biological processes, such as cell proliferation, regulation of cell survival, and modulation of immune functions. IFNs can be divided into three major subfamilies: type I IFNs (comprising mainly IFN-β and over 20 subtypes of IFN-α, -ε, and -ω), type II IFNs (IFN-γ), and the recently identified type III IFNs (IFN-λ) (2, 3) that comprise four members in human (IFN-λ1/IL-29, IFN-λ2, IFN-λ3/IL-28A-B, and IFN-λ4) and two in mice (IFN-λ2/IL-28A and IFN-λ3/IL-28B, while IFN-λ1 is a pseudogene interrupted by a stop codon). IFN-λ2 and IFN-λ3 are highly related and have 96% sequence identity, while IFN-λ1 shares 81% sequence identity with IFN-λ2 and IFN-λ3 (4).

The gene and protein structure of IFN-λ2 and -λ3 share little homology to those of type I IFNs (15%) (4); but they exert remarkably overlapping functions. The heterodimeric receptor for IFN-λ is named IFNLR (or IL-28R), and comprises the specific subunit interferon lambda receptor 1 (IFNLR1, also known as IL-28R1) plus the IL-10R2 subunit that is common to many type II cytokines (such as IL-10, IL-22, IL-24, and IL-26). Once IFNLR is engaged, IFN-λ activate an antiviral response that is very similar to the one triggered by type I IFNs (5). In fact, both engage a similar JAK–STAT pathway, with the only difference that IFN-λ can also use the adaptor JAK2 (6). Both cytokine families also induce IFN-stimulated gene (ISG) transcription, and both confer protection against viral infections (5). This overlap in functions raises the question of why two distinct but similar IFN systems have been maintained throughout evolution, considering that these two systems separated as far back in evolution as did amphibians, reptiles, and birds (7).

The main distinction between the two IFN systems has to do with the tropism between expression of the cytokine and its specific receptors. Myeloid cells at mucosal sites express both type I IFNs and IFN-λ in response to viral as well as bacterial ligands (8–11). However, type I IFN and IFN-λ production are regulated differently. Stimulation of plasma membrane toll-like receptor (TLR) (such as TLR2 and TLR5), both in myeloid and epithelial cells, selectively induces IFN-λ, and not type I IFN, mRNA expression. Moreover, activation of TLR5 has recently been proved to be essential for the induction of IFN-λ upon *Salmonella* encounter (9). Also, cells of epithelial lineage, both in the gut (12) and in the liver (13), preferentially produce IFN-λ over type I IFNs in response to viral ligands. In particular, while both IFNs are induced downstream of pattern recognition receptor and mitochondrial antiviral signaling protein (MAVS), the production of IFN-λ is favored subsequent to activation of the MAVS that reside in peroxisomes (6, 14). The abundance of peroxisomes in cells of epithelial lineage could explain the tropism of IFN-λ production (13).

Other than the tropism of IFN-λ production, the selective expression of the receptor governs the tropism of IFN-λ response. The receptor for type I IFNs (which comprises receptor subunits IFNAR1 and IFNAR2) is expressed in virtually every cell type, while expression of the IFNLR1 receptor is much more specific, and is believed to be most abundant in cells of epithelial origin that are present at barrier surfaces (15). This pattern of expression, along with the recently documented non-redundant role of IFN-λ in protecting against virus infection at mucosal sites [e.g., at the intestinal barrier (12, 16–18) and in the lung (19)], suggest a model in which IFN-λ represents an epithelial cytokine that protect mucosal surfaces without activating widespread and possibly nocuous immune responses, while type I IFNs represent a more general and potent system that is activated once the mucosal barrier is broken. However, recent findings challenge the view that IFN-λ is primarily an epithelial cytokine, describe IFN-λ’s ability to directly and indirectly modulate immune cell functions and document the expression of IFNLR1 on immune cells; they also document that among immune cells, neutrophils express IFNLR1 and directly respond to IFN-λ, in the setting of viral infections (19) as well as other forms of acute inflammation (20–22). IFN-λ reportedly also interferes with the function of NK cells (23, 24), and favors the skewing of T cell activation toward type I (rather than type II) responses, by modulating DC functions (25). While the study of immunomodulatory effects of IFN-λ is still in its infancy—in part due to a lack of specific tools such as good antibodies against IFNLR1—a new role for IFN-λ in shaping the mucosal immune response is emerging. In this review, we critically examine recent literature on the role of IFN-λ in immune cells, differentiating between a direct IFN-λ effect on specific cell types and possible indirect phenomena; we also evaluate what is known about how IFN-λ participates in the control of mucosal immune responses.

## Modulation of Immune Cell Functions by IFN-λ

### Neutrophils

Neutrophils are the first line of defense of the immune system: following pathogen invasion or tissue injury, these cells are quickly and massively recruited to barrier sites, where they protect the host by killing invading pathogens *via* a very rapid release of toxic mediators, independent of *de novo* protein synthesis (26). At later stages, neutrophils regulate the inflammatory response, either passively by undergoing apoptosis and turning off their toxic potential, or actively by secreting anti-inflammatory cytokines and lipidic mediators (27). The ability of these cells to potently kill bacteria is also accompanied by the necessary evil of tissue damage, since many of the toxic mediator released, such as reactive oxygen species (ROS) and proteases, are unable to discriminate between host and pathogen cells. Given the tropism of IFN-λ production to mucosal sites and the complex crosstalk between epithelial cells and neutrophils at mucosal surfaces (28, 29), it is remarkable that among murine immune cells, neutrophils express IFNLR1 at the highest level (19–21). Murine neutrophils express IFNLR1 at very high levels (19–21, 30) that are comparable to those in colonic epithelial cells (20) and in epithelial cells from the lung (19). Human neutrophils have also been found to express IFNLR1 at higher levels as compared to lymphocytes (30) and upregulate its expression following treatment with pro-inflammatory agents such as LPS (20), or after encounter with *Aspergillus fumigatus* (30). In addition to the high levels of receptor expression, mouse and human neutrophils also respond to IFN-λ stimulation (19–21, 30), and activate the canonical JAK–STAT pathway, that leads to phosphorylation of STAT1, STAT2, and STAT3 (21, 30) and induces upregulation of ISGs at levels similar to those induced by type I IFNs (19, 20). Surprisingly, in addition to the canonical ISG response induced downstream of the JAK–STAT pathway, IFN-λ also down-modulates tissue-damaging, transcription-independent responses such as production of ROS, granule mobilization (20), release of neutrophil extracellular traps (NETs) (22), and cellular migration (21); while cytokine production in response to inflammatory stimuli, phagocytosis, and apoptosis is not affected by IFN-λ (20).

Irina Udalova and colleagues were the first to report that neutrophils respond to IFN-λ (21), and that treatment of neutrophils with IFN-λ *in vitro* leads to activation of the JAK–STAT pathway and STAT1 phosphorylation; they also first described the ability of IFN-λ to regulate pro-inflammatory neutrophil functions. In arthritic mice treated with recombinant IFN-λ, they observed a defect in neutrophil migration to the inflamed joint; this defect was attributed to the capacity of IFN-λ to directly inhibit neutrophil migration. Also in an air pouch model of acute inflammation, and when neutrophil migration toward leukotrien B4 was assessed *in vitro*, the cells exhibited a defect in migration: fewer neutrophils were recovered in the air pouch in the presence of IFN-λ, and a shorter Euclidean distance was traveled by neutrophils treated with IFN-λ *in vitro* (21).

More recently, we showed that IFN-λs (but not type I IFNs) are able to regulate a non-translational signaling pathway that diminishes ROS production by neutrophils as well as degranulation following activation of the cells with pro-inflammatory stimuli, but that it does not alter cytokine production induced by inflammatory stimuli or phagocytosis (20). We additionally demonstrated that IFN-λ inhibits degranulation and decreases ROS production even when *de novo* protein synthesis is inhibited with cycloheximide, or when STAT1 or STAT3 are genetically ablated or pharmacologically inhibited. Inhibition of all JAK kinases, or specific inhibition of JAK2, which is involved only in IFN-λ signaling (and not in type I IFN responses) (6, 14) impairs the ability of IFN-λ to inhibit ROS production and degranulation (20). Neutrophils treated with IFN-λ are nevertheless able to phagocytose both opsonized and non-opsonized *E. coli*, and to produce cytokines in response to LPS. Human neutrophils appear to have similar regulating mechanisms: treatment with IFN-λ reduces the ability of these cells to produce ROS (20), and also impairs their ability to generate NETs in an *in vitro* model of thromboinflammation, wherein neutrophils are incubated with activated platelets in the presence of IFN-λ (22). IFN-λ treatment also inhibits NET generation in response to platelet-derived inorganic polyphosphate (polyP) and interferes with the ability of polyP to inhibit mTOR activation and induce the autophagy marker LC3, which is a requisite for NET release (31). IFN-λ, thus, profoundly influences neutrophil non-transcriptional functions and engages a pathway that is independent of the canonical JAK–STAT pathway and does not rely on *de novo* protein synthesis. In contrast to the transcriptional responses, these characteristics are not shared with type I IFNs and seem to specifically target the potent cytotoxic responses that can threaten mucosal integrity.

**Figure 1:**
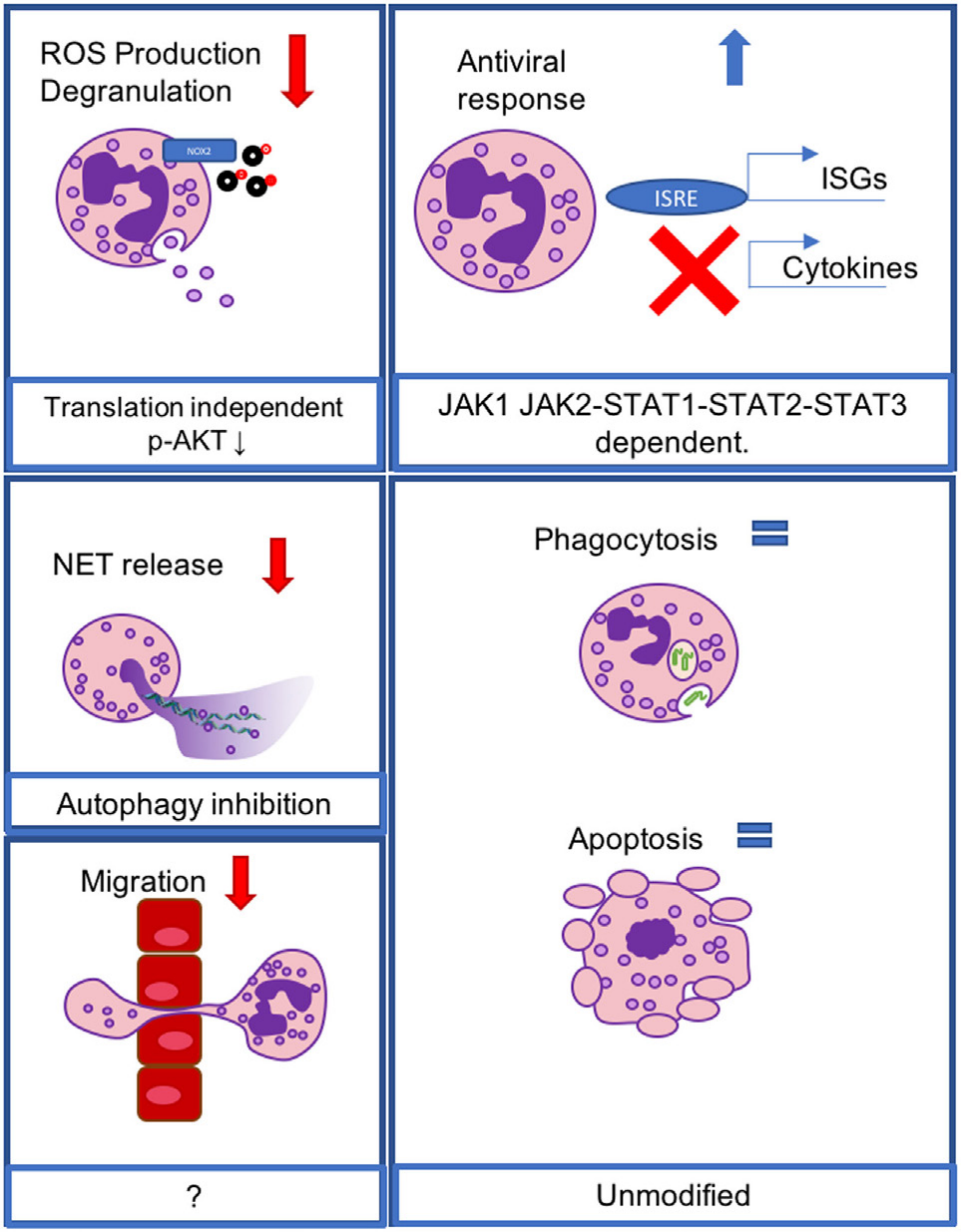
IFN-λ modulates neutrophil functions at the transcriptional and non-transcriptional levels. Reactive oxygen species production and degranulation are regulated at a non-translational level, involving AKT inhibition (upper left), neutrophil extracellular trap release is inhibited *via* inhibition of autophagy (middle left), and neutrophil migration is inhibited *via* an unknown mechanism (lower left). Transcriptional antiviral responses lead to the induction of IFN-stimulated genes, but do not mediate cytokine production, and act through a JAK1- and JAK2-dependent, STAT1, -2, -3-dependent mechanism (upper right). Phagocytosis and apoptosis are not affected (lower right).

As previously described for epithelial cells (2, 3, 5, 32), IFN-λ induces a transcriptional response remarkably similar to that of type I IFNs. So far, no genes have been identified that are selectively upregulated by IFN-λ (and not by type I IFNs), and the upregulation of antiviral ISGs is largely overlapping; however, IFN-λ (as opposed to IFN-α) is unable to directly induce upregulation of pro-inflammatory cytokines, such as TNF, IL-1β, and IL-6, or chemokines, such as CCL2 and CXCL1. The influence of IFN-λ on neutrophils appears, thus, to be anti-inflammatory. Indeed, IFN-λ is able to down-modulate nocuous neutrophil functions—such as the production of toxic mediators or the production of NETs—without interfering with the capacity of these cells to engulf pathogens, or to orchestrate the inflammatory response *via* cytokine secretion (Figure 1). The importance of such regulation of neutrophil functions has been documented *in vivo* following viral infections and also in inflammatory pathologies. In fact, when IFNLR1 is depleted specifically in neutrophils, mice are more susceptible to a sublethal dose of influenza virus infection and present a higher viral load, higher number of leukocytes in the BAL, and higher levels of expression of inflammatory cytokines (19). Notably, when low doses of virus are used for infection, IFNLR expression is required both in epithelial cells and in neutrophils to confer maximum protection. In fact, mice with a conditional ablation of IFNLR1 in pulmonary epithelial cells or in neutrophils only partially recapitulate the total knock-out phenotype (19).

Interferon-λ also influences neutrophil functions during acute inflammation in the gut mucosa. We and others have described a protective role for IFN-λ in a mouse model of DSS-induced colitis (20, 33, 34). In fact, IFNLR1^−/−^ mice are more susceptible to the induction of colitis than are wild-type mice and present a more severe disease phenotype, which is characterized by shorter colons, greater weight loss, more severe histological damage, and augmented oxidative stress (20). This effect is entirely dependent on the action of IFN-λ on immune cells, because chimeras in which only radio-resistant cells are IFNLR1^−/−^, and mice that harbor a deletion of IFNLR1 specific to epithelial cells are equally sensitive to DSS administration as are their wild-type counterparts (20).

By contrast, bone marrow chimeras in which IFNLR1 is depleted only in cells of hematopoietic origin, and mice with conditional depletion of IFNLR1 expression restricted to neutrophils, recapitulate the aggravated phenotype of IFNLR1^−/−^ mice. Notably, both chimeras deleted in the hematopoietic compartment, neutrophils specific IFNLR1^−/−^ mice and total IFNLR1^−/−^ mice have a more severe oxidative stress transcriptional signature in the colon epithelium, when compared to their wild-type counterparts. These data strongly suggest that the control exerted by IFN-λ on neutrophil ROS production is pivotal to protect the intestinal mucosa during acute inflammation (20). In the absence of an active viral or bacterial infection, the source of tonic IFN-λ signaling is represented by the commensal virome. In fact, while depletion of intestinal viruses aggravates colitis in wild-type mice (20, 35) as well as in mice that are deficient in type I IFN signaling (20), IFNLR1^−/−^ mice phenocopy WT mice that are depleted of intestinal viruses in that they are insensitive to treatment with antiviral drugs. In particular, alteration of the intestinal virome in humans that are similar to the alteration obtained in mice treated with antiviral drugs is associated with ulcerative colitis and Crohn’s disease (36, 37).

It was recently shown that IFN-λ action on neutrophils can also protect the host during fungal infections (30). In a model of invasive aspergillosis, both IFNLR1^−/−^ and mice bearing neutrophil-specific depletion of IFNLR1 succumb faster after pulmonary infection with *A. fumigatus* and present an aggravated disease, with higher CFUs recovered from the lungs and more severe invasion as measured by histology. Curiously, neutrophils deficient for IFNLR1 had reduced intracellular ROS levels when stained *ex vivo*. This phenotype was recapitulated in neutrophils deficient for STAT1 suggesting that, during fungal infections, IFN-λ-dependent STAT1 activation mediates a transcriptional program that protects the host. While early, translation independent, regulation of neutrophil function by IFN-λ suppresses ROS production and degranulation in response to inflammatory stimuli, during fungal infections, STAT1-dependent action is critical for the activation of neutrophil functions *in vivo*. The apparent contrast between the two mechanisms can be explained by the differential regulation of neutrophil biology in response to different stimuli. Moreover, while immediate responses, such as ROS production and degranulation, are not typically transcriptionally regulated, the optimal expression of NADPH enzymes during neutrophil development could contribute to the protective effect of IFN-λ against fungi. Indeed, in our hands, when neutrophils were stimulated *in vitro* with *C. albicans* hyphae, ROS were produced both in the absence and in the presence of recombinant IFN-λ (our *unpublished data*). Altogether, these data suggest that IFNLR1-stimulation is not necessary to induce ROS production by neutrophils upon fungal encounter *in vitro* but that, *in vivo*, IFN-λ can contribute to prime neutrophils during a stage of differentiation that could not be recapitulated *in vitro*.

Finally, the inhibitory activity of IFN-λ on neutrophils can also be exploited therapeutically: in fact, IFN-λ administration is protective in pulmonary infections with influenza virus (19), during DSS colitis (20) and in an inflammatory setting such as rheumatoid arthritis (21) or a mouse model of vascular injury (22), where IFN-λ is not produced naturally.

### Dendritic Cells (DCs)

Conventional mouse DCs and human plasmacytoid DCs (pDCs) express low levels of IFNLR1 yet respond to IFN-λ stimulation. In mice, DCs that are derived from the lung express low levels of IFNLR1 (25). Despite these low levels of expression, the central role of DCs at the crossroads between adaptive and innate immunity makes their responses to IFN-λ highly significant. Koltsida and colleagues report that DCs stimulated with IFN-λ, despite responding poorly in terms of ISG induction, are nonetheless able to upregulate T-bet and produce higher levels of IL-12 following LPS stimulation. In the same conditions, they also fail to upregulate OX40L and assume a Th1-polarizing phenotype (25). Indeed, when DCs sorted from the lungs of mice infected with a replication-defective adenovirus expressing IFN-λ under the CMV promoter—or from mice that are treated with recombinant IFN-λ—are used to stimulate T cell polarization *in vitro*, they favor Th1 skewing. This ability of IFN-λ to induce the skewing of T cell responses is particularly relevant in a model of allergic airway disease (25). In fact, IFNLR1^−/−^ mice present a more severe disease phenotype, with elevated production of type II cytokines, a higher histopathological score, and increased eosinophilic infiltration in the BAL. Moreover, when IFN-λ is administered—either directly or *via* an IFN-λ-producing adenovirus—mice are protected from allergic airway disease (25). Also, adoptive transfer of DCs purified from mice treated with IFN-λ-producing adenovirus confers protection. Early reports also suggest that when DCs are stimulated with IFN-λ, they acquire a regulatory phenotype and promote FOXP3^+^ Treg proliferation (38), and that T cell responses can, thus, be skewed toward a Th1 phenotype *in vitro* (39). These data strongly support a role of IFN-λ-stimulated DCs in skewing T cell responses *in vivo*, and underscore the need to further investigate how IFN-λ affects DCs (25).

As mentioned above and recently reviewed (40), human pDCs serve an important role in IFN-λ biology. Human pDCs express IFNLR1 and are able to produce as well as respond to IFN-λ (40–42). When stimulated with IFN-λ, they induce the canonical JAK–STAT pathway (43, 44) and upregulate low levels of ISG transcription (43–45). IFN-λ also influences pDC-specific functions: in particular, it can stimulate pDCs to produce type I IFNs and induce the expression of low levels of TNF (44). Moreover, IFN-λ acts synergistically with IL-3 to hyperactivate pDCs and induce higher levels of inflammatory cytokines (45). Treatment of pDCs with IFN-λ also influences the activation status of pDCs, inducing an upregulation of CD80 and CD86. The functional significance of these regulations remains to be determined: while some researchers claim that IFN-λ inhibits the ability of pDCs to activate T cells (42), the enhancement of pDC activation suggests that IFN-λ stimulates pDCs and enhances their capacity to combat viral infections.

While the ability of IFN-λ to influence the activity of DCs is intriguing and could have a substantial effect on how DCs govern innate and adaptive responses, more work is needed to clarify the specific response of DCs to IFN-λ. The discovery of new non-transcriptional pathways induced by IFN-λ should elucidate whether non-transcriptional responses are active in DCs and help reveal additional specific effects of IFN-λ on DCs. But while scattered reports in the literature link IFN-λ to the skewing of T cells toward a Th1 phenotype (46), the expression of IFNLR in T cells and the responsivity of T cells to IFN-λ has not been formally established; this suggests that the influence of this IFN on T cell functions *in vivo* represents indirect effects that require activation of DCs.

### NK Cells

Emerging evidence documents that IFN-λ affects NK cell activity *in vivo* (23, 24). NK cells are believed to be essential for IFN-λ-mediated protection against influenza virus (24), against tumor growth (23), and in a model of LPS-induced or cecal-ligation puncture (CLP)-induced septic shock (23). However, whether IFN-λ can act directly on NK cells is debated (47–49). Smyth and colleagues (50) report low levels of IFNLR1 expression on mouse NK cells, and to date, there is no evidence of a direct response of NK cells to IFN-λ; in fact, treatment of NK cells with IFN-λ does not activate STAT1 phosphorylation, nor does activate ISG expression (23). However, despite the lack of receptor expression on NK cells and the lack of responsiveness of these cells to IFN-λ *in vitro*, a model of acute endotoxemia shows that NK cells derived from IFNLR1^−/−^ spleens have defective IFN-γ production, and IFNLR1^−/−^ mice are partially protected from lethal doses of LPS or in a CLP model of sepsis, in a IFN-γ-dependent manner. Together, these observations point to an indirect effect of IFN-λ on NK cells. While NK cells transferred from INFLR1^−/−^ mice into Rag^−/−^ γc^−/−^ mice are also defective in the production of IFN-γ after LPS treatment (23), this does not exclude the possibility that IFNLR1^−/−^ NK cells have defects in differentiation/development. Observations on a recent model of influenza virus infection support this notion: administration of IFN-λ (by continuous overexpression *via* hydrodynamic gene delivery) protected mice from the viral infection, and influenced NK cell differentiation; indeed, NK cells in these mice exhibited a more mature phenotype and proliferated at a higher rate. However, these authors also claimed that NK cells express extremely low levels of IFNLR1, and they attributed the observed phenotype to the expression of IFNLR1 on myeloid cells. Notably, depletion of phagocytes by administering clodronate liposomes abolishes the protective effect of IFN-λ (24).

While the above findings unequivocally establish that NK cell functions are modified by IFN-λ *in vivo*, they also strongly suggest that NK cells can be instructed by other cell types that directly respond to IFN-λ stimulation. DCs and neutrophils—the two cell types that do express IFNLR1 and respond to IFN-λ—can influence NK cell functionality *in vivo*. In fact, DCs activate NK cells by secreting cytokines, such as IL-2, IL-18, and IL-12; and DCs also present IL-15 to NK cells in an IFN-β-dependent manner (51–56). It will be important to test in the future the hypothesis that, similarly to type I IFNs (19), IFN-λ could also directly induce low levels of IL-15 that are presented to NK cells. In the same model of airway allergic inflammation that revealed IFN-λs ability to influence DC-mediated skewing of the immune response, it was shown that NK cells preferentially produced IFN-γ and that they were protective against airway inflammation (57). While a direct activity of IFN-λ on NK cells for the observed protection cannot be excluded, the striking similarity of the two models implicates DCs in both skewing NK cell activation and inducing IFN-γ production.

Neutrophils also profoundly influence the functions of NK cells. Consistent with the model in which IFN-λ regulates NK cell maturation, defects in NK cell terminal differentiation and survival were observed in congenitally neutropenic mice and in mice depleted of neutrophils, as well as in patients with neutropenia (57). Also, ROS produced by human neutrophils inhibit NK cell functions *in vitro* (58). The ability of IFN-λ to suppress ROS production and to counteract this inhibition feedback can potentially explain the increased activation of NK cells in the presence of IFN-λs. Some early *in vivo* studies support the hypothesis of a crosstalk between neutrophils and NK cells that governs the antitumoral activity of IFN-λ. In fact, when IFN-λ is administered *via* retroviral transduction into a mouse fibrosarcoma cell line, it is effective in controlling tumor growth, but this protective effect is lost when either NK cells or neutrophils are depleted (59). While IFN-λ undeniably influences NK cell functions *in vivo*, the phenotypes observed might be ascribed to unexplored modulation of NK cell functions by neutrophils or DCs. However, the emergence of non-transcriptional roles for IFN-λ on neutrophils opens up the possibility that similar overlooked non-transcriptional pathways are active in NK cells.

### Other Cell Types

Reports of other cell types expressing IFNLR1 and responding to IFN-λ stimulation exist in the literature. In particular, human B cells have been shown to express IFNLR1 (43, 60, 61) and respond to IFN-λ by upregulating ISGs (61). While the functional role of IFN-λ in B cells is still open for investigations, early pieces of evidence suggest that, similar to type I IFNs, IFN-λ augments TLR-mediated activation of B cells.

Scattered reports describing a role of IFN-λ in human macrophage activation also exist. In particular, IFN-λ can protect human monocyte-derived macrophages from HIV infection (62, 63) and treatment of human monocyte-derived macrophages with IFN-λ augments the production of pro-inflammatory cytokines following stimulation with LPS or R848 (64).

## Conclusion

**Figure 2:**
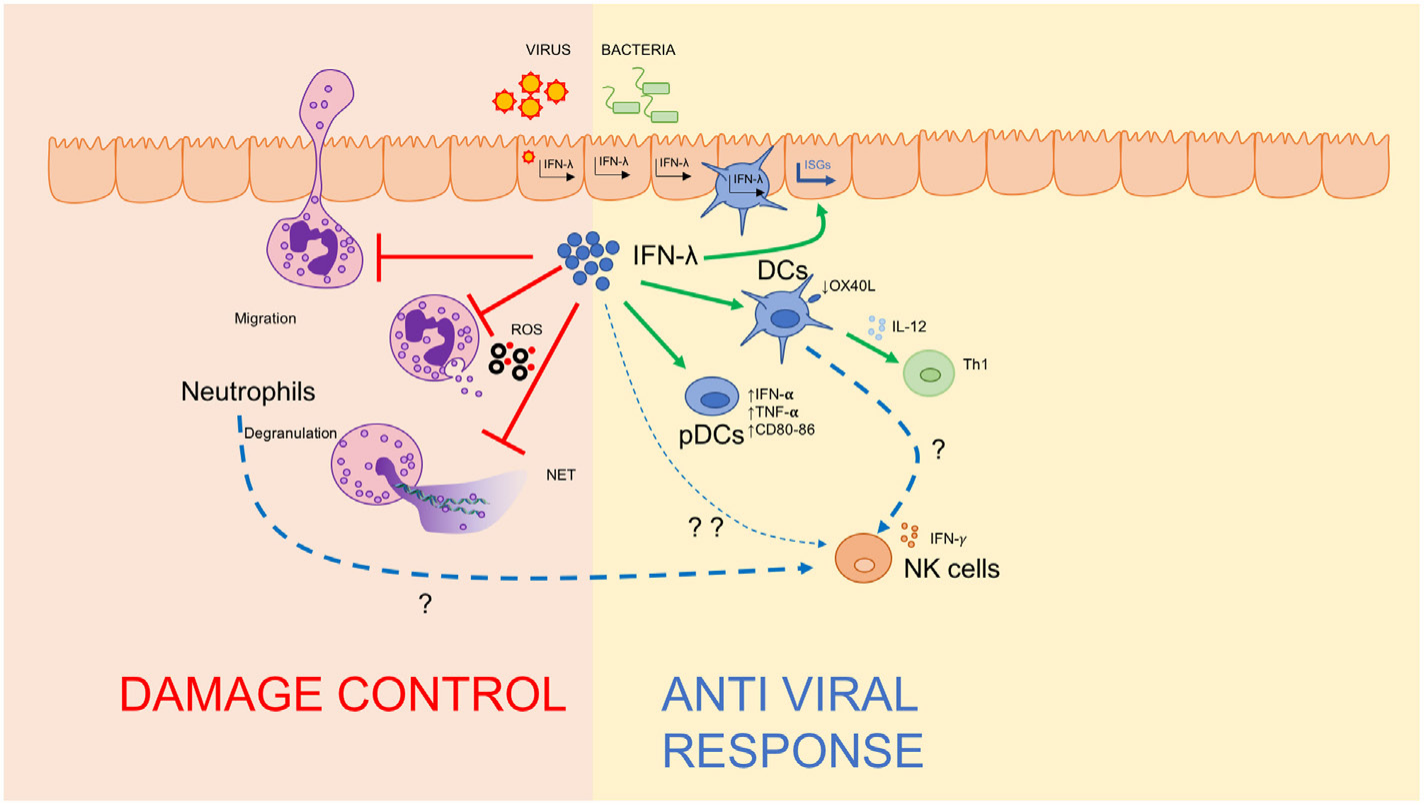
IFN-λ regulates the mucosal inflammatory process. Schematic depiction of IFN-λ’s ability to regulate immunity at mucosal sites by amplifying the antiviral response *via* directly stimulating dendritic cells and plasmacytoid DCs (right), and dampening damage-inducing neutrophil functions to maintain mucosal integrity (left).

Historically, IFN-λ has been recognized as an epithelium-specific cytokine that affects antiviral responses in epithelial cells; however, a growing body of literature supports a critical role for these IFNs in influencing the modulation of immune responses. The action of IFN-λ on immune cells is now configured in a model wherein this cytokine represents the first line of defense of mucosal surfaces. In fact, IFN-λ has non-redundant functions in conditions such as low viral loads (19), or when the epithelial layer is preferentially affected (12): under these conditions, IFN-λ acts directly on epithelial cells to exert local antiviral activity and on DCs to skew the T cell response toward an antiviral Th1 response; IFN-λ also acts directly or indirectly on NK cells to potentiate their activation and protect against viruses. At the same time, IFN-λ also serves important functions in neutrophils, inhibiting tissue-damaging events, such as ROS production, degranulation, and NET formation, without impairing cytokine production or pathogen engulfment. Indeed, IFN-λ activity on neutrophils does not impair, but enhances, responses to pathogenic fungi (30). This modulation of neutrophil activities is pivotal for protecting the mucosae from excessive damage and for maintaining the integrity and barrier functions of epithelia at mucosal sites. IFN-λ is, thus, deemed to be a mucosal cytokine whose evolutionary role is to precede activation of type I IFN, eliminate invading pathogens at mucosal sites without compromising their barrier functions, and limit dissemination of the pathogen (Figure 2). If the pathogen spreads and reaches the underlying tissues, a more potent inflammatory response orchestrated by type I IFNs is needed, but comes at the cost of extensive tissue damage. Such protective activity is also relevant in the absence of a viral infection: tonic IFN-λ production induced by commensal viruses protects the colon mucosa during experimental colitis by dampening neutrophil responses, and administration of IFN-λ is protective in a number of inflammatory settings such as allergic airway diseases, or arthritis. Such evidence of immunomodulatory roles for IFN-λ *in vivo* highlights that these cytokines have additional, as yet unexplored roles in the stimulation of immune cells.

However, support for a direct role for IFN-λ in the modulation of immune functions is fragmented. This is in part due to the lack of biological tools such as specific antibodies against IFNLR1 and the existence of a splicing variant of IFNLR1 in humans that gives rise to a secreted protein with decoy functions (65), which further complicate the correlation of IFNLR1 expression and IFN-λ responsiveness. The translation of findings based on mouse models to human biology is further complicated by the apparent different pattern of expression of the IFNLR1. Indeed, while pDCs and B cells express IFNLR1 and respond to IFN-λ stimulation in humans, the same cell types are not responsive to IFN-λ in mice. Also, while both murine and human neutrophil express the IFNLR1, it is still a matter of discussion if and how inflammatory stimuli and differentiation status of these cells can influence IFNLR1 expression. Despite these confounds, recent reports have uncovered the immune-modulating properties of IFN-λ, as well as new specific non-translational pathways that further differentiate its action from that of type I IFNs. These new insights will pave the way toward an in-depth understanding of the physiological role of these cytokines and will help in exploring the unappreciated functions of IFN-λ in the context of immune cells.

## Author Contributions

All authors listed have made a substantial, direct, and intellectual contribution to the work and approved it for publication.

## Conflict of Interest Statement

The authors declare that the research was conducted in the absence of any commercial or financial relationships that could be construed as a potential conflict of interest.
